# The microbiome of the oral mucosa in irritable bowel syndrome

**DOI:** 10.1080/19490976.2016.1162363

**Published:** 2016-03-10

**Authors:** Nicolaas H. Fourie, Dan Wang, Sarah K. Abey, LeeAnne B. Sherwin, Paule V. Joseph, Bridgett Rahim-Williams, Eric G. Ferguson, Wendy A. Henderson

**Affiliations:** a Division of Intramural Research, National Institute of Nursing Research, National Institutes of Health, DHHS, Bethesda, MD, USA; b National Institute on Minority Health and Health Disparities, National Institutes of Health, DHHS, Bethesda, MD, USA

**Keywords:** irritable bowel syndrome, microbiome, mucosa, overweight, oral, visceral pain

## Abstract

Irritable bowel syndrome (IBS) is a poorly understood disorder characterized by persistent symptoms, including visceral pain. Studies have demonstrated oral microbiome differences in inflammatory bowel diseases suggesting the potential of the oral microbiome in the study of non-oral conditions.

In this exploratory study we examine whether differences exist in the oral microbiome of IBS participants and healthy controls, and whether the oral microbiome relates to symptom severity.

The oral buccal mucosal microbiome of 38 participants was characterized using PhyloChip microarrays. The severity of visceral pain was assessed by orally administering a gastrointestinal test solution. Participants self-reported their induced visceral pain. Pain severity was highest in IBS participants (P = 0.0002), particularly IBS-overweight participants (P = 0.02), and was robustly correlated to the abundance of 60 OTUs, 4 genera, 5 families and 4 orders of bacteria (r^2^ > 0.4, P < 0.001). IBS-overweight participants showed decreased richness in the phylum Bacteroidetes (P = 0.007) and the genus *Bacillus* (P = 0.008). Analysis of β-diversity found significant separation of the IBS-overweight group (P < 0.05). Our oral microbial results are concordant with described fecal and colonic microbiome-IBS and -weight associations. Having IBS and being overweight, rather than IBS-subtypes, was the most important factor in describing the severity of visceral pain and variation in the microbiome. Pain severity was strongly correlated to the abundance of many taxa, suggesting the potential of the oral microbiome in diagnosis and patient phenotyping. The oral microbiome has potential as a source of microbial information in IBS.

## Introduction

Irritable bowel syndrome (IBS) is a syndrome characterized by chronic, persistent symptoms, for which treatment outcomes are notoriously inconsistent.^1^ Integral to the symptom criteria and severity of IBS are chronic visceral pain, visceral hypersensitivity, and altered bowel habits. It is thought that the microbiome plays an integral role in IBS^2^ and is an important target of intervention to relieve IBS symptoms.^3^

Microbiome research in gastrointestinal (GI) disorders and diseases, including IBS, usually use stool or GI mucosa (collected via biopsy) as the primary substrates in which the microbiome is characterized and described. General trends among several GI conditions, such as inflammatory bowel disease (IBD) and IBS indicate a tendency toward decreased microbial richness (i.e. number of OTUs or phylotypes in a community) and diversity (i.e., variation in community structure) and differences in the abundance of certain microbes.^2,4-7^ Interestingly several studies have described similar trends and differences in the richness and abundance of the *oral* microbiota in inflammatory GI conditions^8-10^ as well as non-oral-non-GI diseases.^11,12^ Microbiota in the oral cavity are exposed to regular physical and chemical process (e.g. when ingesting different foods and beverages, or brushing and flossing), these regular environmental perturbations has resulted in a relatively robust and stable microbiome.^13^ The oral microbiome as characterized from various oral substrates appears to be stable within an individual over the short- (days)^14,15^ and long term (years).^16^ The oral microbiome varies less within and between individuals^17^ than the microbiome from other body regions, including the gut.^14^ A comprehensive comparison of microbial communities across different body sites, including the oral cavity and the gut, found that microbial community types of the mouth and the gut were predictive of each other.^18^ This association does not suggest that the oral cavity harbors the same bacteria as the colon, but that perturbations in the intestinal or colonic bacterial communities are reflected in oral bacterial communities.^18^ This phenomenon has been further demonstrated in an animal model of colitis.^9^

Disease associated perturbations in the oral microbiome could be significant as an indicator or diagnostic of systemic dysbiosis and/or specific pathological conditions or risks. Oral health^19^ and variation in the oral microbiome^20^ has been associated with several disease conditions, including IBD (n = 114 and 59),^8,10^ colitis (n = 102),^9^ celiac disease (n = 26),^21^ obesity (n = 543),^22^ leukemia (n = 26),^23^ arthritis (n = 44),^19,24^ and in atherosclerosis (n = 30)^25^ the abundance of certain oral microbial taxa was correlated to plasma cholesterol levels.^25^ Evidence shows that successful periodontal treatment, and by inference the restoration of a healthy oral microbial ecology, improves endothelial function,^26,27^ systemic inflammation,^26^ and glycemic control^28^ related to non-oral conditions.^29^ This suggests that acute, and perhaps subtle, perturbations in the oral microbiome may have systemic effects^11^ or be sensitive to systemic dysregulation of the host's biology. Although, perturbations in the gut microbiome in IBS have and continue to be described,^5,30-32^ *oral* microbial perturbations in IBS, and microbial variation in relations to *symptom severity* remain largely unexplored, although associations between *fecal* microbes and pain^33^ and flatulence severity in IBS patients have been reported.^32^

It remains unclear how the oral microbiota may affect systemic and/or non-oral biology and pathology. Oral microbes are capable of producing toxic and mutagenic metabolites (e.g., alcohol to acetaldehyde)^34^ to which the oral and GI tract is exposed. Oral bacteria can colonize distant GI sites,^11^ or translocate across the epithelial barrier to colonize and infect non-oral systems or organs (e.g. joints and heart).^12^ Microbes could also directly modulate host inflammatory and cell signaling pathways as a ligand source.^35^ Regardless of the exact mechanisms linking the oral microbiome with the gut microbiome and host biology; a growing body of research indicates that the oral cavity is a potential source of microbiome information relevant to non-oral conditions, including IBS. In cases where patients may be uncomfortable with providing stool samples or unable to provide a stool sample at the time of a consultation, or when biopsies represent an unnecessary risk or expense, oral substrates may be a useful minimally invasive source of microbial information with diagnostic potential. Here we perform a preliminary and exploratory analysis, to assess whether the oral microbiome differs between participants with IBS and healthy controls, as has previously been demonstrated in studies of IBD,^8-10^ and whether variation in oral microbes relate to symptom severity, specifically visceral sensitivity and pain. Specifically we compared the buccal mucosa adherent microbiome of participants with IBS to healthy controls and compared the results to previous studies linking the oral microbiome to GI conditions and to the general IBS microbiome literature. We focus specifically on the resident oral mucosa adherent microbiome which is intimately associated with the host epithelium and likely interacts directly with the host's biology.^36,37^

We also recognize that IBS is a symptom defined condition and symptom severity may differ widely among individuals with IBS. The variation in the severity of IBS symptoms may provide a more nuanced interpretation of biological data than symptom categories. We therefor also examine whether the oral microbiome varies with the severity of IBS symptoms, specifically visceral pain, which we induce through an experimental protocol. Differences in the oral microbiome related to IBS and IBS symptom severity would suggest the potential of the buccal mucosa adherent microbiome as a source of information in the study of, diagnosis of and even the treatment and monitoring of IBS and related functional GI disorders. The results of our investigation provide the basis for future research aimed at expanding research on the oral microbiome in functional GI disorders and symptoms.

## Results

### Severity of induced visceral pain (IVP)

**Figure 1.**
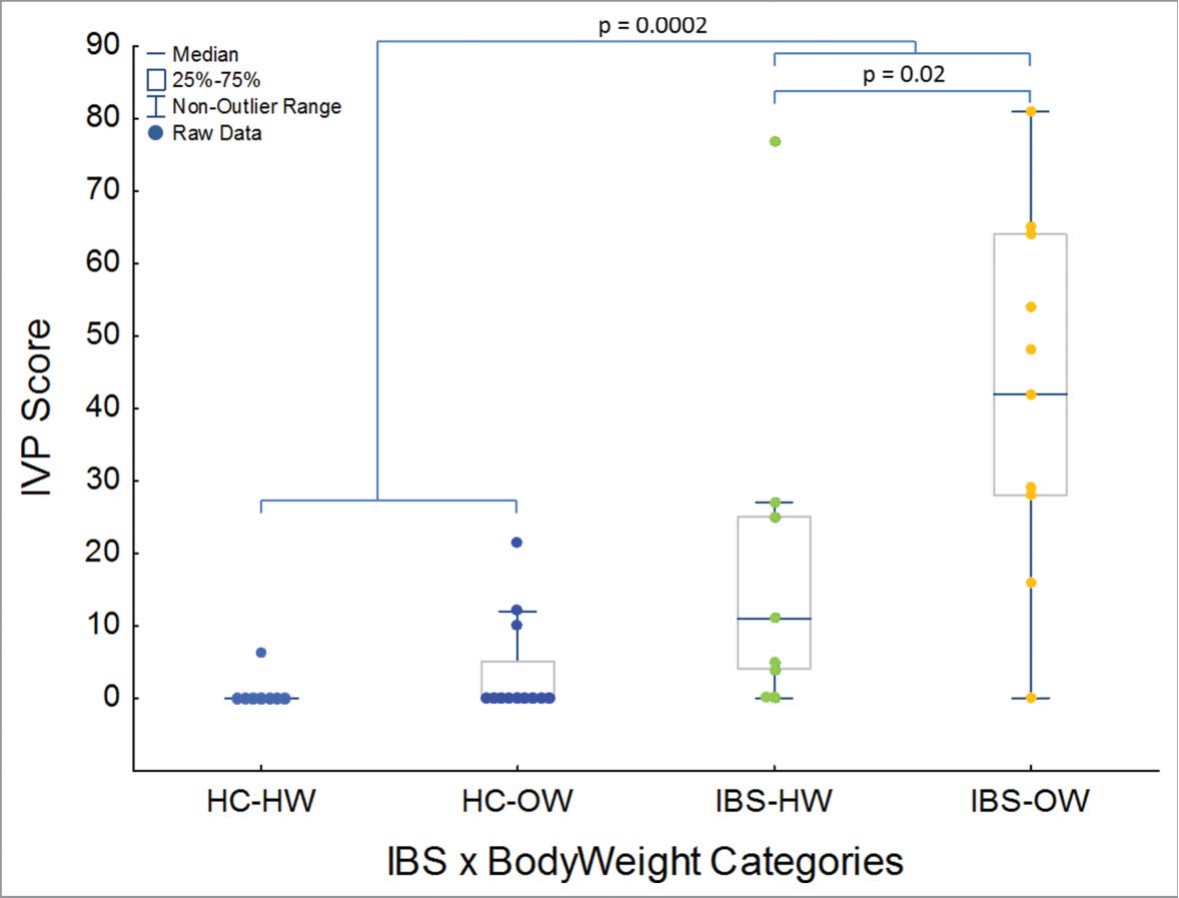
Boxplot showing the prevalence of IVP among IBS and bodyweight groups, and severity of induced visceral pain (IVP) among the 4 groups (HC-HW: n = 8, HC-OW: n = 11, IBS-HW: n = 9, IBS-OW: n =10) of participants. HW = Healthy Weight, OW = Overweight, HC = Healthy Control

Twenty percent of healthy controls (HC) reported IVP in response to the test solution (Mean HC IVP Score = 2.5 ± 5.7; range: 0 to 21.6 on a scale of 0 to a 100) whereas 85% of IBS participants reported IVP (Mean IBS IVP Score = 30.8 ± 26.3; range: 0 to 81) in response to the test solution (Table 1). IBS and the interaction between IBS and body weight significantly (IBS – MWU: p = 0.0002, Weight – MWU: p > 0.05, IBS x Weight – KW: P = 0.0001, Fig. 1) explained IVP severity. Of the 4 HCs which reported IVP in response to the test solution 3 were overweight (Fig. 1). Overweight IBS participants reported significantly higher IVP scores than healthy weight IBS participants (MWU: P = 0.02, Fig. 1). IVP did not differ among IBS subtypes, or between the sexes, nor did the interaction between sex and weight or IBS yield significant differences in IVP (MWU and KW, P > 0.05). Age also did not explain variation in IVP (Pearson's correlation, P > 0.05).

**Table 1.** Clinical characteristics of study cohorts. M = Male, F = Female, BMI = Body Mass Index, IVP = Induced Visceral Pain.

	IBS group	Healthy Controls
Gender (M/F)	8/12	8/12
Age	28 ± 6.9	28 ± 7.3
BMI	27.5 ± 7.4	27.6 ± 6.2
BMI group, *n (%)*		
*Normal Weight*	9 (45)	8 (40)
*Overweight*	11 (55)	12 (60)
Induced Visceral Pain (IVP)	30.8 ± 26.3	2.5 ± 5.7
Incident of IVP, %	85	20
IBS Subtype*, n (%)*		
*Diarrhea*	7 (35)	N/A
*Constipation*	9 (45)	N/A
*Mixed/Undetermined*	4 (20)	N/A

*Note.* *Data is presented as mean ± SD

### Microbiome and pain severity

**Figure 2.**
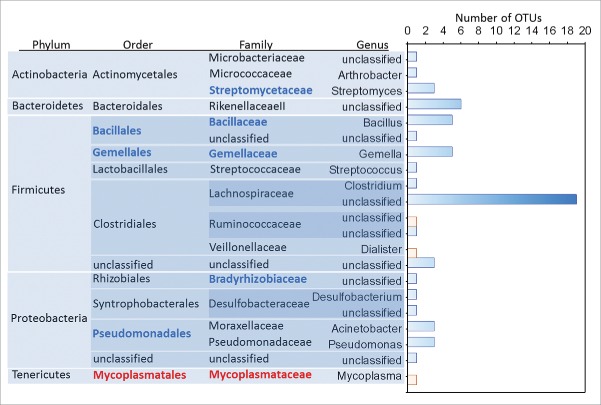
Bar chart showing the number of OTUs (x-axis) grouped by genus, and their taxonomic relationships that were negatively (blue) and positively (red) correlated to the severity of the IVP (n = 38) reported by those participants that reported pain in response to the test solution. Higher order taxonomic groups which significantly correlated to IVP are in bold font in blue (negative correlation) and red (positive correlation).

**Figure 3.**
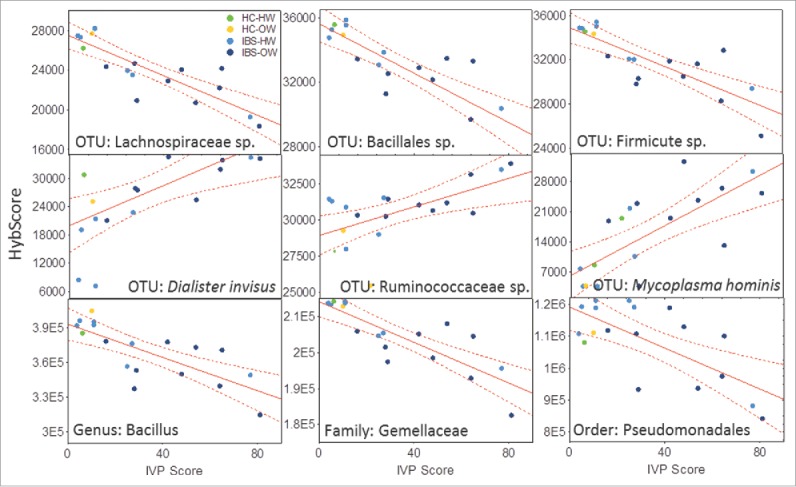
Scatter plots showing the relationship (line) with 95% confidence interval (perforated bands) between the abundance (hybrid score) on the x-axis and IVP (n = 18) (y-axis) for the 3 most negatively correlated OTUs - an unspecified Lachnospiraceae sp (r = −0.85), Bacillales sp. (r = −0.83) and Firmicute sp (r = −0.82), the 3 most positively correlated OTUs – *Dialister invisus* (r = 0.66), a unspecified Ruminococaceae sp (r = 0.69), and *Mycoplasma hominis* (r = 0.75) and a selection of correlated higher order taxonomic groups – the genus *Baccilus* (r = −0.76), the family Gemmellaceae (r = −0.83) and the order Pseudomonadales (r = −0.69). IBS-bodyweight categories are identified on the plots and show greater pain and distinct microbial abundance in IBS-overweight participants (n = 9, dark blue). See Supplementary Table 1 for a full list of all correlations. HW = Healthy Weight, OW = Overweight, HC = Healthy Control.

**Figure 4.**
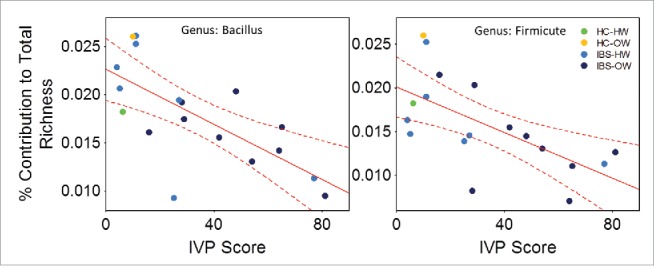
Scatter plots showing the relationship (line) with 95% confidence interval (perforated bands) between the richness and the severity of induced visceral pain (IVP) (n =18) in the genus *Bacillus* (r = −0.70) and an unidentified genus of Firmicute (r = 0.64). IBS-bodyweight categories are identified on the plots and show greater pain and lower richness in IBS-overweight participants (n = 9)(dark blue dots). HW = Healthy Weight, OW = Overweight, HC = Healthy Control

The abundance of 60 OTUs (Pearson's correlation, n = 18, r = −0.85 to 0.75, FDR corrected P = 0.003), 4 genera (*Gemella*-*Bacillus*-*Streptomyces*-*Desulfobacterium*, Pearson's correlation, r = −0.7 to −0.83, FDR corrected P = 0.001), 5 families (Gemellaceae-Bacillacea-Streptomycetaceae-Bradyrhizobiaceae-Mycoplasmataceae, Pearson's correlation, r = −0.67 to −0.83, FDR corrected P = 0.002) and 4 orders (Gemellales-Bacillales-Pseudomonadales-Mycoplasmatales, Pearson's correlation, r = −0.7 to −0.83, FDR corrected P = 0.002) of bacteria correlated significantly to the IVP scores reported by participants that experienced pain in response to the test solution (Figs. 2 and 3, see supplementary Table 1 for complete table of correlations and statistics). Four OTUs correlated to IVP had species level descriptions, these were *Acinobacter johnsonii* (r = −0.67, Family: Moraxellaceae), *Gemella sanguinis* (−0.79, Family: Gemellaceae), *Mycoplasma hominis* (r = 0.75, Family: Mycoplasmataceae) and *Dialister invisus* (r = 0.66, Family: Veillonellaceae) (Fig. 3). The richness of the genus *Bacillus* (n = 18, r = −0.70) and an unclassified genus of Firmicute (r = −0.64) decreased significantly as the severity of IVP increased across participants which reported pain (Pearson's correlation, FDR corrected P = 0.008, Fig. 4).

### Microbial richness and abundance in IBS and body weight

PCoA of unweighted and weighted UniFrac distances based on all OTUs did not show clear differentiation between bodyweight, IBS, IBS-subtype. The IBS-overweight category did show some separation along the first 2 PCoA axes of PCoA analyses of both unweighted and weighted UniFrac distances. PCoA identified the healthy control number 25 as an extreme outlier. Comparisons among categories using the weighted UniFract dissimilarity matrix and the ADONIS method to test for differences among categories found that the IBS-overweight group differed significantly at the P < 0.1 level (weighted UniFrac distance matrix, ADONIS, P = 0.084) from all other groups combined. No other significant or near significant divergence in overall bacterial community richness (unweighted UniFrac) or abundance (weighted UniFrac) distance metrices between categories were observed.

**Figure 5.**
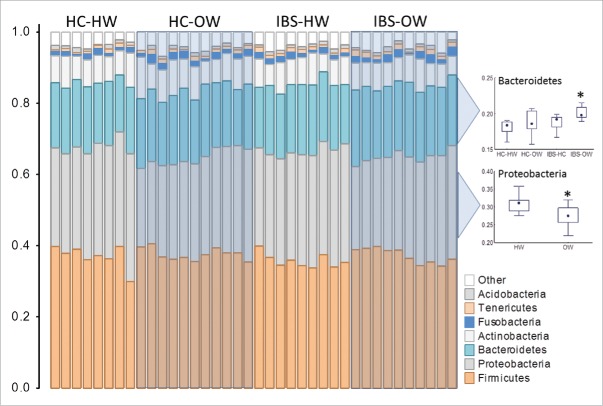
Richness profiles of the top 10 richest phyla in the oral mucosa of IBS-bodyweight categories. Individuals are represented along the horizontal axis and the relative richness of phyla (expressed as a % of total richness) are on the vertical axis. Healthy weight participants (HC-HW: n = 8, IBS-HW: n = 9) are grouped in the unshaded areas and overweight participants (HC-OW: n = 11, IBS-OW: n = 10) are grouped in the shaded boxes. Differences in richness for selected phyla are presented in the box (25th to 75th percentile) and whisker (non-outlier range) plot. Significantly different groups are indicated with an asterisk*. HW = Healthy Weight, OW = Overweight, HC = Healthy Control

Over all bacterial richness (i.e. number of OTUs) did not differ between categories (sex, body weight, IBS, IBS x body weight and IBS-subtype) or correlate with the severity of IVP. However analysis of the top 10 richest taxa at the genus, family, order, class and phylum level revealed specific taxa that differed between healthy weight (n = 19) and overweight groups (n = 19) (Fig. 5), and the interaction between IBS and body weight (Fig. 5), while variability the richness of other taxa correlated with IVP severity (Fig. 4). Members of an unclassified genus of Proteobacteria, the genus *Pseudomonas* (Welch test, FDR corrected P = 0.01), an unclassified family of Proteobacteria, the family Pseudomonadaceae, (Welch test, FDR corrected P = 0.015), an unclassified class of Proteobacteria and the class Gammaproteobacteria (Welch test, FDR corrected P = 0.01), the phylum Proteobacteria (Welch test, FDR corrected P = 0.005) were detected at significantly lower proportions in overweight individuals. OTUs of the family Veillonellaceae were proportionally over represented in overweight participants (Welch test, FDR corrected P = 0.015). Proteobacteria OTUs were present at lower proportions in overweight healthy individuals (ANOVA, FDR corrected P = 0.01, Tukey's HSD, P = 0.03) and tended to be lower in IBS-overweight participants (Tukey's HSD, P = 0.07); whereas overweight IBS participants (n = 10) had a significantly higher proportion of Bacteroidetes OTUs than normal weight healthy controls (n = 8) (ANOVA, FDR corrected P = 0.01, Tukey's HSD, P = 0.007) and normal weight IBS participants (n = 9) (Tukey's HSD, P = 0.07, Fig. 5).

**Figure 6.**
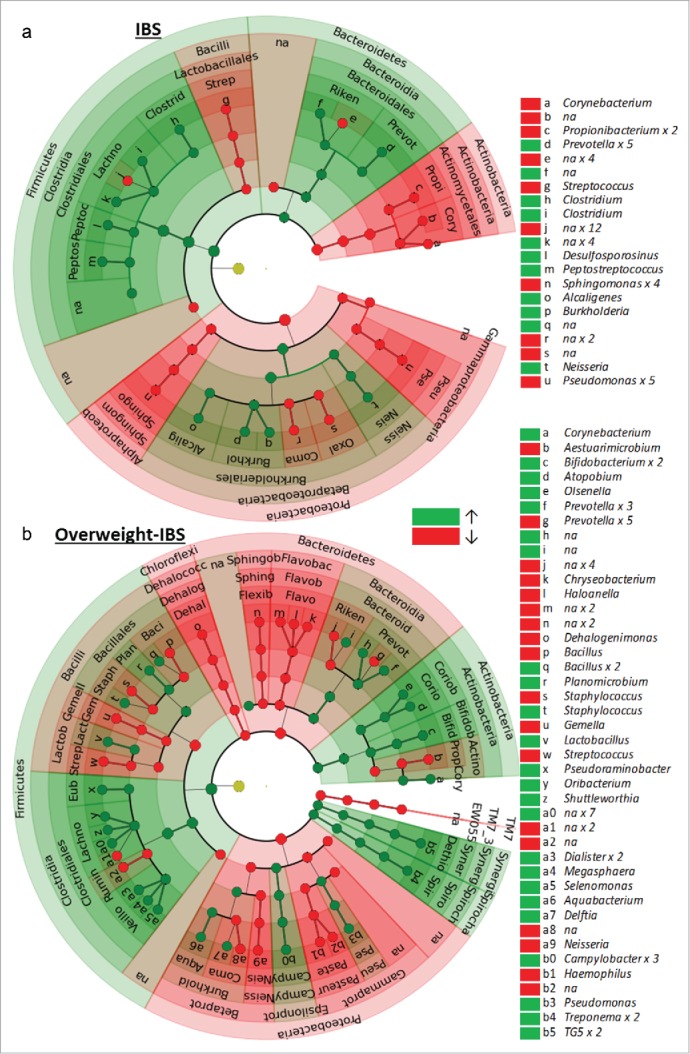
A and b Circular cladogram showing the taxa (up to the genus level) that were more (green) and less (red) abundant in (a) IBS participants vs. Healthy Controls, and in (b) overweight IBS participants vs. Healthy Controls. See Supplementary Tables 2 and 3 for more detailed list of taxa and statistics.

**Figure 7.**
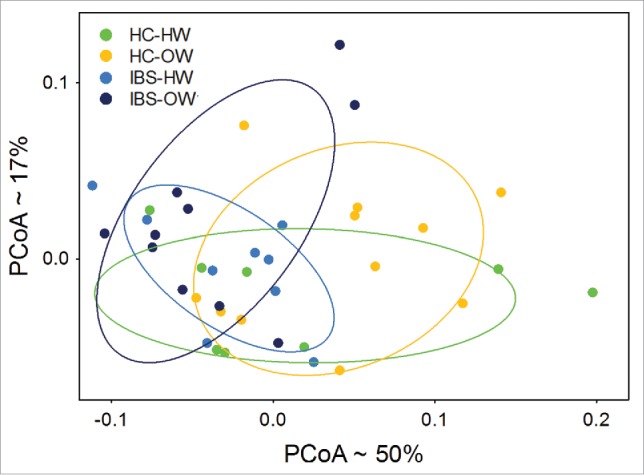
Principal Coordinate Analysis of weighted UniFrac distances (n =38) computed from OTUs differentially expressed between IBS and HC groups showing significant (ADONIS test, P < 0.05) separation of the IBS-overweight group from other groups. Ellipses represent the 95% intervals for each IBS-body weight category. The approximate proportion (%) of variance explained by each principal coordinate axis is reported in the axis label. HW = Healthy Weight, OW = Overweight, HC = Healthy Control.

Fifty six OTUs showed significant differential abundance (Welch test, P < 0.05, not FDR adjusted, Fig. 6a, Supplementary Table 2) between healthy controls and IBS participants. PCoA of the weighted UniFrac distances computed from these 56 OTUs showed moderate separation between IBS and HC groups, and between IBS-body weight groups along the first 2 PCoA axes (Fig. 7). Group comparisons using the weighted UniFrac distances found significant differences between IBS (n = 19) and HC (n = 19) groups (ADONIS, P = 0.003) and among IBS-bodyweight groups (ADONIS, P = 0.01). The IBS overweight group (n = 10) differed significantly from (ADONIS, P = 0.039) all other groups combined.

**Figure 8.**
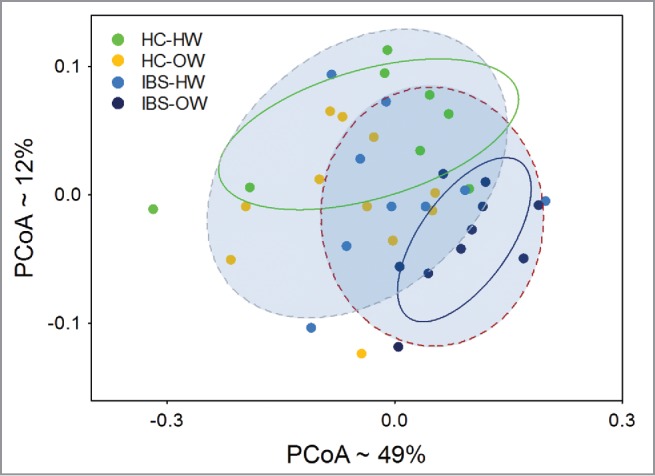
Principal Coordinate Analysis of weighted UniFrac distances (n =38) computed from OTUs differentially expressed between overweight IBS participants and HCs showing significant (ADONIS test, P < 0.05) separation of the IBS group from healthy controls, and IBS-overweight group from all other groups. The ellipses (solid lines = HC-HW and IBS-OW, perforated lines = IBS and HCs) represent the 95% intervals for each IBS-body weight category. The approximate proportion (%) of variance explained by each principal coordinate axis is reported in the axis label. HW = Healthy Weight, OW = Overweight, HC = Healthy Control.

Further analysis focusing on the IBS overweight group found 74 OTUs differed significantly (Welch test, P < 0.05, not FDR adjusted, Fig. 6b, Supplementary Table 3) in abundance between IBS overweight participants and healthy controls. The abundance of these 74 OTUs was used to compute a weighted UniFrac distance matrix. PCoA of the matrix shows both the healthy weight healthy controls and IBS overweight groups as distinct clusters separated along the y-axis (Fig. 8). Overweight healthy controls and healthy weight IBS participants do not differentiate from each other and show substantial overlap with the healthy weight healthy controls. ADONIS tests further reveal that significant differences exist between IBS and healthy controls (ADONIS, P = 0.005), and among IBS-bodyweight groups (ADONIS, P = 0.003). Specifically the IBS overweight group (ADONIS, P = 0.002) differ significantly from all other groups combined. Comparisons of α diversity found no differences.

## Discussion

Overweight IBS participants in our study reported significantly more severe pain in response to the gastrointestinal test solution, and those healthy controls that reported IVP tended to be overweight individuals. Being overweight is a known risk factor for a broad range of pain conditions^38,39^ including visceral pain. The severity or sensitivity to pain has previously been positively associated with BMI.^40-42^ These initial findings indicated that a weight effect may be present throughout our subsequent analyses.

Specifically, overweight individuals that also suffered from IBS reported the highest pain intensity in response to the test solution, followed by healthy weight participants with IBS. Healthy controls reported the least or no pain in response to the test solution. This IBS-overweight mediated pain gradient intensity was significantly correlated to a number of oral bacterial taxa identified from the buccal mucosa. Overweight IBS participants either had very high or very low abundances (and sometimes richness) of IVP correlated microbes (Figs. 3 and 4). An increased abundance of a *Desulfobacterium* sp., a *Prevotella* sp*., Dialister invisus* and *Mycoplasma hominis* were associated with IBS and IVP severity. The oral abundance of members of sulfate reducing bacteria (e.g., *Desulfobacterium*) have been previously associated with gastritis and periodontal disease,^43^ while fecal overabundance is associated with IBD.^44^ Elevated abundance of *Prevotella*^45^ and members of the Veillonellaceae (*Dialister* sp.)^6,46^ are known in patients with IBS.^47,48^ Members of these taxa are also known to be overexpressed in the oral substrates of individuals suffering from IBD.^10^ A Ruminocaccaceae OTU was also positively associated with IVP severity; fecal members of the family have also been positively associated with abdominal pain frequency and intensity in IBS.^32,33^ We find a strong positive correlation between *Mycoplasma hominis* and pain severity, this microbe is not usually associated with GI conditions but has been positively associated with several pain and inflammatory conditions such as fibromyalgia, pelvic inflammatory disease and arthritis.^49^

The majority of microbial associations with IBS and IVP were negative. Decreased abundance could be observed at higher taxonomic groupings (e.g. Order and Family) suggesting a trend of lowered abundance across many OTUs. Members of the order Bacillales, in particular the genus *Bacillus* are well known as probiotic organisms and are used in the treatment of IBS.^50^ *Gemella sanguinis* has been described at lower abundances in the oral mucosa of patients with Crohn's Disease,^10^ whereas the bacterium *Actinobacter viridans* is known to have probiotic properties,^51,52^ the abundance of these microbes co-vary negatively with pain severity. It is also interesting to note decreased abundance in a relatively large number of Lachnospiraceae, other members of the Clostridiales order, and unclassified Bacteroidetes OTUs in relation to IVP severity. Fecal Lachnospiraceae and unknown Bacteriodes OTUs have previously been found to negatively associate with the frequency of recurrent abdominal pain in IBS.^33^ Similar decreases in butyrate producing phylotypes have been described in IBS.^32,46^ The Clostridiales contain an abundance of short-chain-fatty-acid (SCFA) producing members, particularly microbes that produce butyric acid.^53-55^ Butyrate is known to have protective anti-inflammatory and anti-cancer effects.^56,57^

Categorical comparisons of microbial richness and abundance yielded less robust differences between participants with IBS and healthy controls. Body weight, specifically being overweight remained a significant driver of categorical differences in the microbiome but the interaction of this variable reduces statistical power and results should not be over interpreted. Our analyses found that the IBS-overweight group was often the most distinct in its microbial makeup from all other groups. In analyses of microbial distance metrices (weighted UniFrac) the interaction between bodyweight and IBS was a significant factor explaining the distribution of individuals relative to one another. Overweight participants appeared to exhibit decreased richness in key taxa. The phylum Proteobacteria, in particular the class and members (Family: Pseudomonadaceae, genus: *Pseudomonas*) of the class Gammaproteobacteria showed decreased richness in the overweight cohort, while a greater richness of OTUs and abundance of specific OTUs (*Dialister* sp, *Megasphaera* sp.) belonging the family Veillonellaceae was observed in overweight group. Proteobacteria in the gut has been shown to decrease in response to high fat diets^58^ and the Gammaproteobacteria have been found to increase with weight-loss.^59^ This pattern is recapitulated in the oral cavity where Proteobacteria appear to be more abundant in the saliva of healthy individuals than overweight individuals,^60^ which is congruent with our findings in oral mucosa. An increased relative abundance in oral^22,61,62^ and gut^63^ Veillonellaceae is associated with increased carbohydrate and fat consumption, and obesity. The observed overabundance of *Prevotella* sp (gut *Prevotella* associated with high carbohydrate and simple sugar diets)^64^ and *Treponema* sp. (oral Treponema sp associated with simple sugars)^65^ and decreased abundance of *Gemella* sp. in overweight participants in our study has also previously been described.^22,66^ Although we do not have detailed dietary information for paticipants; inferring high-fat-high-carbohydrate diets for overweight participants based on differential microbial expression is consistent with diet-microbiome/obesity-diet associations reported in the literature. Similarly the observed oral overabundance in OTUs belonging to the *Prevotella*,^33,47,48,67^ Bacteriodetes,^68^ decreased abundance of Microbacteriales, Micrococaceae (Fig. 2), *Corynbacterium*, *Propionibacterium*, Oxalobacteraceae^68^ (Fig. 6a and b, Supplementary Table 2) and Lachnospiraceae in IBS has also been observed in fecal samples in IBS. Some incongruence between oral and gut microbiota are to be expected as the oral bacteria differ in aspects of taxonomy, environment and function from those in the gut, non-the-less the observed IBS associated differences in the oral microbiome are similar to those observed in the gut.

Furthermore, our results indicate an interaction between weight and IBS. Overweight IBS participants had the most distinct oral microbiome and microbial differences, presenting microbial characteristics of both obesity and gastrointestinal diseases such as IBD. The metabolic and inflammatory dysregulation associated with unhealthy body weight and associated high-fat-high-carbohydrate diets could result in/or compound hypersensitivity, hyperalgesia and other IBS symptoms, resulting in a mosaic-overweight-IBS-microbial difference. Studies specifically designed and powered to research this association are needed to better understand microbial perturbations. The oral microbiome may also reflect the effects of other systemic processes, such as sub-clinical inflammation, which has previously been suggested in IBS, or even be the source of GI dysbiosis and symptoms.^20^ Regardless of the mechanism driving the observed microbial differences and variation, and their association with symptom severity, IBS and obesity; the buccal mucosa appears to provide condition relevant microbial information.

Notwithstanding the relatively small sample sizes used, our results find that oral buccal mucosal microbiome reflects differences in the microbial ecology of participants with IBS that are overweight. These results emphasize the importance of bodyweight as a factor determining microbial composition and abundance, and perhaps should be considered a significant factor in defining IBS phenotypes and symptom severity. We also show that symptom severity is an important nuance which may help explain variation in the biology underlying IBS. Our study aimed to assess the potential of the oral microbiome, specifically the buccal mucosal adherent fraction, as a source of microbial information relevant to IBS. Our preliminary results suggest the oral microbiome is indeed a promising source of microbial information relevant to the study of IBS, obesity and related symptom severity. The research reported here will be expanded upon to examine the generalizability of the observed patterns.

Although promising, the current investigation has several limitations and our results should be interpreted within these constraints. The current study was designed as an initial assessment and is underpowered to control for/and take all sources of microbial variation into account.

However, unlike the gut microbiome the oral microbiome appears to be more stable and less variable within and between individuals over time.^13-16^ It remains to be examined how responsive the oral microbiome is to transient phenomena (e.g., eating certain foods, oral hygiene etc.) and how this may affect sampling strategies. We speculate that given the oral microbiomes unusual stability (compared to the gut microbiome) and the adaptation of oral biofilms to regular physical and chemical perturbations that it is less sensitive than other GI communities. None-the-less, interpretation of the oral microbiome, as with the gut microbiome, is complicated by a range of anthropogenic confounders (e.g. socio-economic and culturally mediated dietary or hygiene preferences etc.) which should be controlled for as far as is possible, and considered in data analysis and the interpretation of results. The results of this study forms the basis of ongoing research where sample sizes will be expanded and contributing sources of variation can be better understood. Several studies have shown simultaneous dysbiosis of the microbiome, or covariation of the microbiome between different substrates from different anatomical regions of the body,^69-71^ including comparisons of the oral and GI microbiome.^9,18^ The nature and cause of these observations remain poorly understood. We endeavor to collect both fecal and oral samples for future comparisons to better understand if and how the microbiome of these 2 substrates co-varies in IBS. Notwithstanding these limitations, we are encouraged by our results which recover microbial associations with IBS and bodyweight congruent with the results of previous reports, as well as robust novel associations with symptom (visceral pain) severity.

Our findings contribute to a growing body of evidence that the oral microbiome reflects differences associated with GI and other non-oral conditions.^8-12,26^ The oral microbial associations we describe are largely concordant with existing reports of IBS and bodyweight related microbial associations based on observations in stool and GI biopsies. Collection of GI mucosal biopsies is invasive, can be costly, and usually require colonic cleansing, which itself could exacerbate GI symptoms, or may not be indicated in for diagnosis of IBS. Colonic cleansing itself may alter the observed microbiome, whereas stool samples may only poorly reflect the mucosal adherent microbial fraction. Alternative non-invasive, cheap, low risk substrates such as the oral mucosa could play an important role in the microbial characterization, treatment, and monitoring of IBS, associated symptoms and other functional GI conditions.^72^

## Materials and methods

### Study participants

Healthy controls (HC, n = 20) were defined as individuals who did not report any GI symptoms and had no known organic disease. IBS participants (n = 20) were individuals who suffered from chronic visceral pain and altered bowel habits for more than 6 months for which no organic cause had been identified (Rome III).^73^ Based on these information, participants were subtyped as IBS-diarrhea (IBS-D, n = 7), IBS-constipation (IBS-C, n = 9) or IBS-mixed (IBS-M, n = 4). Study participants and healthy controls were as closely matched as possible for gender, race, age, and weight. Participants were classified as normal weight or overweight based on their body mass index (BMI). All IBS participants and HCs were resident within the greater Washington, DC area, which includes surrounding suburbs in Maryland and Virginia. Volunteers that tested positive for active *Helicobacter pylori* and/or *Clostridium difficile* infections were excluded from this study. Table 1 summarizes the demographic profile of the samples as well as clinical variables for participants. In addition, participants were required to fast for 12 hours prior to sampling to control for the transient effects of different foods and beverages on the oral microbiome. The careful selection described here provides some measure of control, as far as possible in a cross-sectional human sample, for socio-demographic and other effects on the oral microbiome.

### Experimental GI stressor

After an initial screening visit that included medical history, physical exam and laboratory evaluation, participants who met inclusion criteria were invited back for a second visit. During the second visit, participants (including controls) described their visceral pain intensity, sensation and location using the Gastrointestinal Pain Pointer (GIPP), an electronic self-report interface,^74^ before and after ingestion of a 100 ml intestinal permeability test solution (sucrose - 10 g/dl, lactulose - 5 g/dl, mannitol - 1 g/dl, and sucralose - 0.1 g/dl) formulated by the NIH pharmacy.^75^ We administered the test solution to induce a visceral pain response, termed induced visceral pain (IVP). Participants reported the intensity of their pain using a virtual dial on an app-like electronic interface, the GIPP, on a 0-100 point scale to which they are blinded. For more information on the GIPP and its validation please refer to Henderson et al. 2015. IVP was measured at 30 minutes prior to ingesting the solution, at the time of ingestion, and at 15, 30, 45, 60, 90, 120, 180, 240, and 300 minute intervals post ingestion. The maximum IVP scores recorded were used for downstream analyses.

### Microbiome profiling

Buccal swabs were collected using a Cytobrush® (CooperSurgical, Berlin Germany). Buccal cells and mucus were mechanically separated in phosphate buffered saline, pelleted and stored at −80^o^C until extraction. DNA was purified from the cells using BiOstic®Bacteremia DNA Isolation Kits (MO-BIO Laboratories, Carlsbad, CA). The manufacturer's instructions were followed without modification.

### *16S* rRNA amplification

The extracted DNA was shipped to SecondGenome for amplification, purification, hybridization, and microarray analysis. SecondGenome processed the samples in a “Good Laboratory Practices” compliant laboratory. Bacterial 16S rRNA was amplified by polymerase chain reaction (PCR) using the 27F.1 (5’-AGRGTTTGATCMTGGCTCAG-3’) forward primer and 1492R.jgi (5’-GGTTACCTTGTTACGACTT-3’) reverse primer.^76^ Thirty-5 cycles of PCR amplification were performed. The amplified PCR product of each sample was purified using a solid-phase reversible immobilization method. The purified PCR products were quantified using an Agilent 2100 Bioanalyszer®. Thirty-nine samples amplified to specification and were moved forward to hybridization. Bacterial 16S rRNA gene amplicons were fragmented, biotin labeled, and hybridized to the PhyloChip™ Array, version G3. PhyloChip arrays were washed, stained, and scanned using a GeneArray® scanner (Affymetrix). Each scan was captured using standard Affymetrix software (GeneChip® Microarray Analysis Suite). The scan for one sample did not pass required QC specifications and was excluded from data analysis. Data for 38 samples were moved forward to data analysis.

### Data processing and analysis

Data processing was completed by SecondGenome Inc. In short; fluorescent intensity was calculated and hybridization scores (HybScore) were derived. Hybridization scores represent the abundance of a given operational taxonomic unit (OTU), the lowest taxonomic unit used in analyses. Abundance was calculated for each successively higher taxonomic classification by summing the HybScores for all OTUs belong to the given higher order taxonomic units. A binary metric for each OTU was computed to determine whether it was considered present or absent in a sample. An OTU was considered present if the positive fraction of a probe for an OTU was greater than or equal to 0.8. The top 10 richest taxa at each taxonomic level were used to compare the differential contribution of each taxon to overall richness between weight and IBS groups (Welch test and ANOVA and Tukey's HSD) and to explore the relationship of variation in the richness of each taxon to IVP scores (Pearson's correlation). The parametric Welch test was employed to compare categories (IBS and Weight) using the abundance data. Pearson's correlation (p < 0.05) was used to examine the relationship between microbial abundance and IVP scores. Untransformed HybScores were used in correlation analysis. The presence and absence of IVP generally reflected the IBS and HC categories respectively (pain = IBS, no pain = HC). Correlation analysis, therefore, largely reflected categorical comparisons. In order to circumvent the bimodal effect of such data on correlations, only data from participants who reported IVP in response to the GI test solution were used. Sample number 25 was excluded from these analyses as an extreme outlier.

The Benjamini-Hochberg procedure was used to control for false discovery rates (FDR). All abundance and presence –absence (relative abundance) profiles were compared in a pair-wise fashion to determine a dissimilarity score (UniFrac distance= based on richness data, Weighted UniFrac = based on abundance data) from which a distance dissimilarity matrix was constructed.^77^ Principal Coordinate Analysis (PCoA) was used to explore the relationship of individual samples to each other in 2 dimensional space using the UniFract and Weighted UniFrac distance matrices. The ADONIS test was used to test for categorical distance in the differences matrices.

Analysis of IVP data was done using non-parametric Mann-Whitney U (MWU) and Kruskal-Wallis ANOVA (KW). Statistical analyses were conducted using R 3.2.2 and STATISTCA 12 (Dell™) statistical packages. Data analysis was performed in collaboration with SecondGenome's data analysis service.

## Supplementary Material

KGMI_A_1162363_Supplementals.zip

## Data sharing statement

Data used to generate the results of this study are available on request from the corresponding author at hendersw@mail.nih.gov

## References

[1] Drossman DA, Camilleri M, Mayer EA, Whitehead WE. AGA technical review on irritable bowel syndrome. Gastroenterology 2002; 123(6):2108-31; PMID:12454866; http://dx.doi.org/10.1053/gast.2002.37095 12454866

[2] Major G, Spiller R. Irritable bowel syndrome, inflammatory bowel disease and the microbiome.” Curr Opin Endocrinol Diabetes Obes 2014; 21(1):15-21; PMID:24296462; http://dx.doi.org/10.1097/MED.0000000000000032 24296462 PMC3871405

[3] Shanahan F, Quigley EMM. Manipulation of the Microbiota for Treatment of IBS and IBD—Challenges and Controversies. Gastroenterology 2014; 146(6):1554-63; PMID:24486051; http://dx.doi.org/10.1053/j.gastro.2014.01.050 24486051

[4] Carroll IM, Ringel-Kulka T, Siddle JP, Ringel Y. Alterations in composition and diversity of the intestinal microbiota in patients with diarrhea-predominant irritable bowel syndrome. Neurogastroenterol Motil 2012; 24(6):521-30; PMID:22339879; http://dx.doi.org/10.1111/j.1365-2982.2012.01891.x 22339879 PMC3975596

[5] Kennedy PJ, Cryan JF, Dinan TG, Clarke G. Irritable bowel syndrome: a microbiome-gut-brain axis disorder? World J Gastroenterol 2014; 20(39): 14105-25; PMID:25339800; http://dx.doi.org/10.3748/wjg.v20.i39.14105 25339800 PMC4202342

[6] Lee KJ, Tack J. Altered intestinal microbiota in irritable bowel syndrome. Neurogastroenterol Motil 2010; 22(5):493-8; PMID:20414959; http://dx.doi.org/10.1111/j.1365-2982.2010.01482.x 20414959

[7] Ponnusamy K, Choi JN, Kim J, Lee SY, Lee CH. Microbial community and metabolomic comparison of irritable bowel syndrome faeces. J Med Microbiol 2011; 60(Pt 6): 817-27; PMID:21330412; http://dx.doi.org/10.1099/jmm.0.028126-0 21330412 PMC3167923

[8] Docktor MJ, Paster BJ, Abramowicz S, Ingram J, Wang YE, Correll M, Jiang H, Cotton SL, Kokaras AS, Bousvaros A. Alterations in diversity of the oral microbiome in pediatric inflammatory bowel disease. Inflamm Bowel Dis 2012; 18(5): 935-42; PMID:21987382; http://dx.doi.org/10.1002/ibd.21874 21987382 PMC4208308

[9] Rautava J, Pinnell LJ, Vong L, Akseer N, Assa A, Sherman PM Oral Microbiome Composition Changes in Mouse Models of Colitis. J Gastroenterol Hepatol 2014; 2(10): 12713. 10.1111/jgh.12713 25180790

[10] Said HS, Suda W, Nakagome S, Chinen H, Oshima K, Kim S, Kimura R, Iraha A, Ishida H, Fujita J, et al. Dysbiosis of salivary microbiota in inflammatory bowel disease and its association with oral immunological biomarkers. DNA Res 2014; 21(1): 15-25; PMID:24013298; http://dx.doi.org/10.1093/dnares/dst037 24013298 PMC3925391

[11] Ahn J, Chen CY, Hayes RB. Oral microbiome and oral and gastrointestinal cancer risk.” Cancer Causes Control 2012; 23(3):399-404; PMID:22271008; http://dx.doi.org/10.1007/s10552-011-9892-7 22271008 PMC3767140

[12] Li X, Kolltveit KM, Tronstad L, Olsen I. Systemic Diseases Caused by Oral Infection. Clin Microbiol Rev 2000; 13(4): 547-58; PMID:11023956; http://dx.doi.org/10.1128/CMR.13.4.547-558.2000 11023956 PMC88948

[13] Zaura E, Nicu EA, Krom BP, Keijser BJ Acquiring and maintaining a normal oral microbiome: current perspective. Front Cell Infect Microbiol 2014; 4:85; PMID:25019064; http://dx.doi.org/19892944 10.3389/fcimb.2014.00085 PMC4071637 25019064

[14] Costello EK, Lauber CL, Hamady M, Fierer N, Gordon JI, Knight R. Bacterial community variation in human body habitats across space and time. Science 2009; 326(5960):1694-7; PMID:19892944; http://dx.doi.org/10.1126/science.1177486 19892944 PMC3602444

[15] Lazarevic V, Whiteson K, Hernandez D, Francois P, Schrenzel J. Study of inter- and intra-individual variations in the salivary microbiota.” BMC Genomics 2010; 11: 523; PMID:20920195; http://dx.doi.org/10.1186/1471-2164-11-523 20920195 PMC2997015

[16] Rasiah IA, Wong L, Anderson SA, Sissons CH. Variation in bacterial DGGE patterns from human saliva: over time, between individuals and in corresponding dental plaque microcosms. Arch Oral Biol 2005; 50(9): 779-87; PMID:15970209; http://dx.doi.org/10.1016/j.archoralbio.2005.02.001 15970209

[17] Wade WG. The oral microbiome in health and disease. Pharmacol Res 2013; 69(1):137-43; PMID:23201354; http://dx.doi.org/10.1016/j.phrs.2012.11.006 23201354

[18] Ding T, Schloss PD. Dynamics and associations of microbial community types across the human body. Nature 2014; 509(7500):357-60; PMID:24739969; http://dx.doi.org/10.1038/nature13178 24739969 PMC4139711

[19] Wolff B, Berger T, Frese C, Max R, Blank N, Lorenz HM, Wolff D. Oral status in patients with early rheumatoid arthritis: a prospective, case-control study. Rheumatology 2014; 53(3): 526-31; PMID:24273047; http://dx.doi.org/10.1093/rheumatology/ket362 24273047

[20] Han YW, Wang X. Mobile microbiome: oral bacteria in extra-oral infections and inflammation. J Dent Res 2013; 92(6):485-91; PMID:23625375; http://dx.doi.org/10.1177/0022034513487559 23625375 PMC3654760

[21] Francavilla R, Ercolini D, Piccolo M, Vannini L, Siragusa S, De Filippis F, De Pasquale I, Di Cagno R, Di Toma M, Gozzi G, et al. Salivary microbiota and metabolome associated with celiac disease. Appl Environ Microbiol 2014; 80(11):3416-25; PMID:24657864; http://dx.doi.org/10.1128/AEM.00362-14 24657864 PMC4018861

[22] Goodson JM, Groppo D, Halem S, Carpino E. Is Obesity an Oral Bacterial Disease? J Dent Res 2009; 88(6):519-23; PMID:19587155; http://dx.doi.org/10.1177/0022034509338353 19587155 PMC2744897

[23] Wang Y, Xue J, Zhou X, You M, Du Q, Yang X, He J, Zou J, Cheng L, Li M, et al. Oral microbiota distinguishes acute lymphoblastic leukemia pediatric hosts from healthy populations. PLoS One 2014; 9(7):e102116; PMID:2502546 25025462 10.1371/journal.pone.0102116 PMC4099009

[24] Yeoh N, Burton JP, Suppiah P, Reid G, Stebbings S The role of the microbiome in rheumatic diseases. Curr Rheumatol Rep 2013; 15(3): 012-0314; http://dx.doi.org/10.1007/s11926-012-0314-y 23378145

[25] Koren O, Spor A, Felin J, Fåk F, Stombaugh J, Tremaroli V, Behre CJ, Knight R, Fagerberg B, Ley RE et al. Human oral, gut, and plaque microbiota in patients with atherosclerosis. Proc Natl Acad Sci U S A 2011; 108(Supplement 1):4592-8; PMID:20937873; http://dx.doi.org/21133981 10.1073/pnas.1011383107 20937873 PMC3063583

[26] Li X, Tse HF, Yiu KH, Li LS, Jin L. Effect of periodontal treatment on circulating CD34(+) cells and peripheral vascular endothelial function: a randomized controlled trial. J Clin Periodontol 2011; 38(2):148-56; PMID:21133981; http://dx.doi.org/10.1111/j.1600-051X.2010.01651.x 21133981

[27] Tonetti MS, D'Aiuto F, Nibali L, Donald A, Storry C, Parkar M, Suvan J, Hingorani AD, Vallance P, Deanfield J. Treatment of Periodontitis and Endothelial Function.” N Engl J Med 2007; 356(9): 911-20; PMID:17329698; http://dx.doi.org/10.1056/NEJMoa063186 17329698

[28] Teeuw WJ, Gerdes VE, Loos BG. Effect of periodontal treatment on glycemic control of diabetic patients: a systematic review and meta-analysis. Diabetes Care 2010; 33(2):421-7; PMID:20103557; http://dx.doi.org/10.2337/dc09-1378 20103557 PMC2809296

[29] Moura Foz A, Alexandre Romito G, Manoel Bispo C, Luciancencov Petrillo C, Patel K, Suvan J, D'Aiuto F. Periodontal therapy and biomarkers related to cardiovascular risk. Minerva Stomatol 2010; 59(5):271-83; PMID:20502435 20502435

[30] Jalanka J, Salonen A, Fuentes S, de Vos WM. Microbial Signatures in Post-Infectious Irritable Bowel Syndrome – Towards Patient Stratification for Improved Diagnostics and Treatment. Gut Microbes 2015; 6:364-9; PMID:26512631 26512631 10.1080/19490976.2015.1096486 PMC4826089

[31] Mayer EA, Savidge T, Shulman RJ. Brain-gut microbiome interactions and functional bowel disorders. Gastroenterology 2014; 146(6): 1500-12; PMID:24583088; http://dx.doi.org/10.1053/j.gastro.2014.02.037 24583088 PMC4114504

[32] Pozuelo M, Panda S, Santiago A, Mendez S, Accarino A, Santos J, Guarner F, Azpiroz F, Manichanh C Reduction of butyrate- and methane-producing microorganisms in patients with Irritable Bowel Syndrome. Sci Rep 2015; 5:12693; http://dx.doi.org/10.1038/srep12693 26239401 PMC4523847

[33] Saulnier DM, Riehle K, Mistretta TA, Diaz MA, Mandal D, Raza S, Weidler EM, Qin X, Coarfa C, Milosavljevic A, et al. Gastrointestinal Microbiome Signatures of Pediatric Patients With Irritable Bowel Syndrome. Gastroenterology 2011; 141(5): 1782-1791; PMID:21741921; http://dx.doi.org/10.1053/j.gastro.2011.06.072 21741921 PMC3417828

[34] Moritani K, Takeshita T, Shibata Y, Ninomiya T, Kiyohara Y, Yamashita Y. Acetaldehyde production by major oral microbes. Oral Dis 2015; 21(6): 748-754; PMID:25809116; http://dx.doi.org/10.1111/odi.12341 25809116

[35] Chinen T, Volchkov PY, Chervonsky AV, Rudensky AY. A critical role for regulatory T cell-mediated control of inflammation in the absence of commensal microbiota. J Exp Med 2010; 207(11):2323-30; PMID:20921284; http://dx.doi.org/10.1084/jem.20101235 20921284 PMC2964571

[36] Slack E, Hapfelmeier S, Stecher B, Velykoredko Y, Stoel M, Lawson MAE, Geuking MB, Beutler B, Tedder TF, Hardt W-Det al. Innate and Adaptive Immunity Cooperate Flexibly to Maintain Host-Microbiota Mutualism. Science 2009; 325(5940):617-20; PMID:19644121; http://dx.doi.org/10.1126/science.1172747 19644121 PMC3730530

[37] Tailford LE, Crost EH, Kavanaugh D, Juge N. Mucin glycan foraging in the human gut microbiome. Front Genet 2015; 6:81; PMID:25852737; http://dx.doi.org/10.3389/fgene.2015.00081 25852737 PMC4365749

[38] Stone AA, Broderick JE. Obesity and Pain Are Associated in the United States. Obesity 2012; 20(7):1491-5; PMID:22262163; http://dx.doi.org/10.1038/oby.2011.397 22262163

[39] Wright LJ, Schur E, Noonan C, Ahumada S, Buchwald D, Afari N. Chronic Pain, Overweight, and Obesity: Findings from a Community-Based Twin Registry. J Pain 2010; 11(7): 628-35; PMID:20338816; http://dx.doi.org/10.1016/j.jpain.2009.10.004 20338816 PMC2892725

[40] Astita RA, Tashani OA, Sharp D, Johnson MI. Argument for the need of investigation of the relationship between body fatness and experimental pain sensitivity. Libyan J Med 2015; 10:28457 ; PMID:26085491; http://dx.doi.org/10.3402/ljm.v10.28457 26085491 PMC4471214

[41] Kim C-H, Luedtke CA, Vincent A, Thompson JM, Oh TH. Association of body mass index with symptom severity and quality of life in patients with fibromyalgia. Arthritis Care Res 2012; 64(2): 222-8; PMID:21972124; http://dx.doi.org/10.1002/acr.20653 21972124

[42] Ravn P, Frederiksen R, Skovsen AP, Christrup LL, Werner MU. Prediction of pain sensitivity in healthy volunteers.” J Pain Res 2012; 5: 313-26; PMID:23055774; http://dx.doi.org/10.2147/JPR.S33925 23055774 PMC3442738

[43] Heggendorn FL, Souza Gonçalves L, Dias EP, Silva Junior A, Galvão MM, Lutterbach MTS. Detection of sulphate-reducing bacteria in human saliva. Acta Odontol Scand 2013; 71(6): 1458-1463; PMID:23638810; http://dx.doi.org/10.3109/00016357.2013.770163 23638810

[44] Loubinoux J, Bronowicki J-P, Pereira IAC, Mougenel J-L, Le Faou AE Sulfate-reducing bacteria in human feces and their association with inflammatory bowel diseases; 2002 10.1111/j.1574-6941.2002.tb00942.x 19709217

[45] Shankar V, Agans R, Holmes B, Raymer M, Paliy O. Do gut microbial communities differ in pediatric IBS and health? Gut Microbes 2013; 4(4):347-52; PMID:23674073; http://dx.doi.org/10.4161/gmic.24827 23674073 PMC3744519

[46] Jeffery IB, O'Toole PW, Öhman L, Claesson MJ, Deane J, Quigley EMM, Simrén M. An irritable bowel syndrome subtype defined by species-specific alterations in faecal microbiota. Gut 2012; 61(7):997-1006; PMID:22180058; http://dx.doi.org/10.1136/gutjnl-2011-301501 22180058

[47] Rajilic-Stojanovic M, Biagi E, Heilig HG, Kajander K, Kekkonen RA, Tims S, de Vos WM. Global and deep molecular analysis of microbiota signatures in fecal samples from patients with irritable bowel syndrome. Gastroenterology 2011; 141(5):1792-801; PMID:21820992; http://dx.doi.org/10.1053/j.gastro.2011.07.043 21820992

[48] Rigsbee L, Agans R, Shankar V, Kenche H, Khamis HJ, Michail S, Paliy O. Quantitative profiling of gut microbiota of children with diarrhea-predominant irritable bowel syndrome. Am J Gastroenterol 2012; 107(11):1740-51; PMID:22986438; http://dx.doi.org/10.1038/ajg.2012.287 22986438

[49] Nicolson GL, Nasralla MY, Franco AR, Meirleir KD, Nicolson NL, Ngwenya R, Haier J Role of Mycoplasmal Infections in Fatigue Illnesses. J Chronic Fatigue Syndrome 2000; 6(3-4): 23-39; http://dx.doi.org/10.1300/J092v06n03_03

[50] Hun L. Original Research: Bacillus coagulans Significantly Improved Abdominal Pain and Bloating in Patients with IBS. Postgrad Med 2009; 121(2):119-24; PMID:19332970; http://dx.doi.org/10.3810/pgm.2009.03.1984 19332970

[51] Ramirez-Chavarin ML, Wacher C, Eslava-Campos CA . Probiotic potential of thermotolerant lactic acid bacteria strains isolated from cooked meat products. Int Food Res J 2013; 20: 991-1000.

[52] Zhurylo OA, Turliun SA, Drozd T. [A biological model study of the effect of Aerococcus viridans on pathogenic bacteria]. Mikrobiol Z 1998; 60(3): 56-62; PMID:9785800 9785800

[53] Charrier C, Duncan GJ, Reid MD, Rucklidge GJ, Henderson D, Young P, Russell VJ, Aminov RI, Flint HJ, Louis P). A novel class of CoA-transferase involved in short-chain fatty acid metabolism in butyrate-producing human colonic bacteria. Microbiology 2006; 152(Pt 1):179-85; PMID:16385128; http://dx.doi.org/10.1099/mic.0.28412-0 16385128

[54] Duncan SH, Barcenilla A, Stewart CS, Pryde SE, Flint HJ). Acetate utilization and butyryl coenzyme A (CoA):acetate-CoA transferase in butyrate-producing bacteria from the human large intestine. Appl Environ Microbiol 2002; 68(10): 5186-90; PMID:12324374; http://dx.doi.org/10.1128/AEM.68.10.5186-5190.2002 12324374 PMC126392

[55] Louis P, Duncan SH, McCrae SI, Millar J, Jackson MS, Flint HJ. Restricted distribution of the butyrate kinase pathway among butyrate-producing bacteria from the human colon. J Bacteriol 2004; 186(7):2099-106; PMID:15028695; http://dx.doi.org/10.1128/JB.186.7.2099-2106.2004 15028695 PMC374397

[56] McIntyre A, Gibson PR, Young GP. Butyrate production from dietary fibre and protection against large bowel cancer in a rat model. Gut 1993; 34(3): 386-91; PMID:8386131; http://dx.doi.org/10.1136/gut.34.3.386 8386131 PMC1374147

[57] Singh N, Gurav A, Sivaprakasam S, Brady E, Padia R, Shi H, Thangaraju M, Prasad PD, Manicassamy S, Munn DH, et al. Activation of Gpr109a, receptor for niacin and the commensal metabolite butyrate, suppresses colonic inflammation and carcinogenesis. Immunity 2014; 40(1):128-39; PMID:24412617; http://dx.doi.org/10.1016/j.immuni.2013.12.007 24412617 PMC4305274

[58] Murphy EF, Cotter PD, Healy S, Marques TM, O'Sullivan O, Fouhy F, Clarke SF, O'Toole PW, Quigley EM, Stanton C, et al. Composition and energy harvesting capacity of the gut microbiota: relationship to diet, obesity and time in mouse models. Gut 2010; 59(12):1635-42; PMID:20926643; http://dx.doi.org/10.1136/gut.2010.215665 20926643

[59] Santacruz A, Marcos A, Warnberg J, Marti A, Martin-Matillas M, Campoy C, Moreno LA, Veiga O, Redondo-Figuero C, Garagorri JM, et al. Interplay between weight loss and gut microbiota composition in overweight adolescents. Obesity (Silver Spring) 2009; 17(10): 1906-15; PMID:19390523; http://dx.doi.org/10.1038/oby.2009.112 19390523

[60] Piombino P, Genovese A, Esposito S, Moio L, Cutolo PP, Chambery A, Severino V, Moneta E, Smith DP, Owens SM, et al. Saliva from Obese Individuals Suppresses the Release of Aroma Compounds from Wine. PLoS One 2014; 9(1):e85611; PMID:24465618; http://dx.doi.org/10.1371/journal.pone.0085611 24465618 PMC3899019

[61] Adler CJ, Dobney K, Weyrich LS, Kaidonis J, Walker AW, Haak W, Bradshaw CJA, Townsend G, Soltysiak A, Alt KW, et al. Sequencing ancient calcified dental plaque shows changes in oral microbiota with dietary shifts of the Neolithic and Industrial revolutions. Nat Genet 2013; 45(4):450-5; PMID:23416520; http://dx.doi.org/10.1038/ng.2536 23416520 PMC3996550

[62] Ercolini D, Francavilla R, Vannini L, De Filippis F, Capriati T, Di Cagno R, Iacono G, De Angelis M, Gobbetti M. From an imbalance to a new imbalance: Italian-style gluten-free diet alters the salivary microbiota and metabolome of African celiac children. Sci Rep 2015; 5: 18571; PMID:26681599; http://dx.doi.org/10.1038/srep18571 26681599 PMC4683525

[63] Lecomte V, Kaakoush NO, Maloney CA, Raipuria M, Huinao KD, Mitchell HM, Morris MJ.Changes in Gut Microbiota in Rats Fed a High Fat Diet Correlate with Obesity-Associated Metabolic Parameters.” PLoS ONE 2015; 10(5):e0126931; PMID:25992554; http://dx.doi.org/10.1371/journal.pone.0126931 25992554 PMC4436290

[64] Wu GD, Chen J, Hoffmann C, Bittinger K, Chen Y-Y, Keilbaugh SA, Bewtra M, Knights D, Walters WA, Knight R, et al. Linking Long-Term Dietary Patterns with Gut Microbial Enterotypes. Science 2011; 334(6052):105-8; PMID:21885731; http://dx.doi.org/10.1126/science.1208344 21885731 PMC3368382

[65] Baumgartner S, Imfeld T, Schicht O, Rath C, Persson RE, Persson GR). The impact of the stone age diet on gingival conditions in the absence of oral hygiene. J Periodontol 2009; 80(5):759-68; PMID:19405829; http://dx.doi.org/10.1902/jop.2009.080376 19405829

[66] Schwiertz A, Taras D, Schäfer K, Beijer S, Bos NA, Donus C, Hardt PD. Microbiota and SCFA in Lean and Overweight Healthy Subjects. Obesity 2010; 18(1):190-5; PMID:19498350; http://dx.doi.org/10.1038/oby.2009.167 19498350

[67] Jalanka-Tuovinen J, Salojarvi J, Salonen A, Immonen O, Garsed K, Kelly FM, Zaitoun A, Palva A, Spiller RC, de Vos WM. Faecal microbiota composition and host-microbe cross-talk following gastroenteritis and in postinfectious irritable bowel syndrome. Gut 2014; 63(11): 1737-45; PMID:24310267; http://dx.doi.org/10.1136/gutjnl-2013-305994 24310267

[68] Ng SC, Lam EF, Lam TT, Chan Y, Law W, Tse PC, Kamm MA, Sung JJ, Chan FK, Wu JC. Effect of probiotic bacteria on the intestinal microbiota in irritable bowel syndrome. J Gastroenterol Hepatol 2013; 28(10): 1624-31; PMID:23800182 23800182 10.1111/jgh.12306

[69] Morgan XC, Tickle TL, Sokol H, Gevers D, Devaney KL, Ward DV, Reyes JA, Shah SA, LeLeiko N, Snapper SB. Dysfunction of the intestinal microbiome in inflammatory bowel disease and treatment. Genome Biol 2012; 13(9): R79; PMID:23013615; http://dx.doi.org/10.1186/gb-2012-13-9-r79 23013615 PMC3506950

[70] Mutlu E, Keshavarzian A, Engen P, Forsyth CB, Sikaroodi M, Gillevet P Intestinal Dysbiosis: A Possible Mechanism of Alcohol-Induced Endotoxemia and Alcoholic Steatohepatitis in Rats. Alcohol Clin Exp Res 2009; 33(10):1836-46; PMID:19645728; http://dx.doi.org/25445885 10.1111/j.1530-0277.2009.01022.x 19645728 PMC3684271

[71] Schmidlin PR, Fachinger P, Tini G, Graber S, Seifert B, Dombrowa S, Irani S. Shared microbiome in gums and the lung in an outpatient population. J Infect 2015; 70(3): 255-263; PMID:25445885; http://dx.doi.org/10.1016/j.jinf.2014.10.005 25445885

[72] Zhang Y, Sun J, Lin C-C, Abemayor E, Wang MB, Wong D. The emerging landscape of salivary diagnostics. Oral Health Dent Manag 2014; 13: 200-10; PMID:24984623 24984623

[73] Drossman DA, Corazziari E, Delvaux M, Spiller R, Talley N, Thompson WG, Whitehead WE Rome III: The Functional Gastrointestinal Disorders. McLean, VA, Degnon Associates, Inc., 2006

[74] Henderson WA, Shankar R, Taylor TJ, Del Valle-Pinero AY, Kleiner DE, Kim KH, Youssef NN. Inverse relationship of interleukin-6 and mast cells in children with inflammatory and non-inflammatory abdominal pain phenotypes. World J Gastrointest Pathophysiol 2012; 3(6): 102-8; PMID:23516176; http://dx.doi.org/10.4291/wjgp.v3.i6.102 23516176 PMC3602438

[75] Del Valle-Pinero AY, Sherwin LB, Anderson EM, Caudle RM, Henderson WA. Altered vasoactive intestinal peptides expression in irritable bowel syndrome patients and rats with trinitrobenzene sulfonic acid-induced colitis. World J Gastroenterol 2015; 21(1):155-63; PMID:25574088; http://dx.doi.org/10.3748/wjg.v21.i1.155 25574088 PMC4284331

[76] DeSantis T, Brodie E, Moberg J, Zubieta I, Piceno Y, Andersen G. High-Density Universal 16S rRNA Microarray Analysis Reveals Broader Diversity than Typical Clone Library When Sampling the Environment. Micro Ecol 2007; 53(3): 371-83; PMID:17334858; http://dx.doi.org/10.1007/s00248-006-9134-9 17334858

[77] Lozupone C, Hamady M, Knight R. UniFrac - An online tool for comparing microbial community diversity in a phylogenetic context. BMC Bioinformatics 2006; 7(1): 371; PMID:16893466; http://dx.doi.org/10.1186/1471-2105-7-371 16893466 PMC1564154

